# A New Target for Amyloid Beta Toxicity Validated by Standard and High-Throughput Electrophysiology

**DOI:** 10.1371/journal.pone.0008643

**Published:** 2010-01-08

**Authors:** Kucku Varghese, Peter Molnar, Mainak Das, Neelima Bhargava, Stephen Lambert, Mark S. Kindy, James J. Hickman

**Affiliations:** 1 NanoScience Technology Center, University of Central Florida, Orlando, Florida, United States of America; 2 Department of Neuroscience, Medical University of South Carolina, Charleston, South Carolina, United States of America; 3 College of Medicine, University of Central Florida, Orlando, Florida, United States of America; Massachusetts General Hospital and Harvard Medical School, United States of America

## Abstract

**Background:**

Soluble oligomers of amyloid beta (Aβ) are considered to be one of the major contributing factors to the development of Alzheimer's disease. Most therapeutic development studies have focused on toxicity directly at the synapse.

**Methodology/Principal Findings:**

Patch clamp studies detailed here have demonstrated that soluble Aβ can also cause functional toxicity, namely it inhibits spontaneous firing of hippocampal neurons without significant cell death at low concentrations. This toxicity will eventually lead to the loss of the synapse as well, but may precede this loss by a considerable amount of time. In a key technological advance we have reproduced these results utilizing a fast and simple method based on extracellular electrophysiological recording of the temporal electrical activity of cultured hippocampal neurons using multielectrode arrays (MEAs) at low concentrations of Aβ (1–42). We have also shown that this functional deficit can be reversed through use of curcumin, an inhibitor of Aβ oligomerization, using both analysis methods.

**Conclusions/Significance:**

The MEA recording method utilized here is non-invasive, thus long term chronic measurements are possible and it does not require precise positioning of electrodes, thus it is ideal for functional screens. Even more significantly, we believe we have now identified a new target for drug development for AD based on functional toxicity of hippocampal neurons that could treat neurodegenerative diseases prior to the development of mild cognitive impairment.

## Introduction

We have demonstrated that high-throughput electrophysiology techniques can be used to measure Amyloid beta (Aβ) toxicity in neurons and that the effects of this toxicity can be reversed by a drug application. In addition, we believe we have identified a new target for drug development for Alzheimer's Disease (AD) that focuses on loss of electrical functionality of the cell that may precede synapse degradation by a considerable period of time. These results support the emerging view that functional impairment of neurons may be more important for the development of AD symptoms than the actual cell death which occurs at later stages of the disease [1], [2].

AD is the most common cause of dementia in the elderly [3]. The hallmarks of this disease consist of senile plaques composed of Aβ, neurofibrillary tangles and extensive neuronal degeneration [4]. Aβ is a 39–43 amino acid peptide derived from the cleavage of a larger protein, Amyloid Precursor Protein (APP), and is toxic to neurons *in vivo* and *in vitro* [5]. The amyloid cascade hypothesis implicates Aβ as having a crucial role in the pathogenesis of AD [6] and as a result is an important therapeutic target. Recent results have implicated soluble aggregates of Aβ for many of the toxic effects of Aβ described in AD [7].

Although it is well known that one of the early hallmarks of AD is marked synaptic degeneration, its cause is only marginally understood [8], [9]. According to the leading theory, soluble oligomers of Aβ have a direct “synaptotoxic” effect at nanomolar concentrations [10], [11]. Other authors have emphasized the success of Memantine, an NMDA antagonist, in moderate and severe cases of AD, and claim that excitotoxicity could play a role in synaptic degeneration [12]–[14]. Our hypothesis is based on the observation that Aβ decreases spontaneous activity of neurons at low concentrations and that this has a deleterious effect on cell functionality without significant cell death. According to established theories, this decreased activity would lead to the automatic elimination of synapses with low activity, but at a later time [15], [16].

It has also been shown that Aβ toxicity can be reversed to varying degrees using anti-amyloidogenic compounds (AACs) such as curcumin [17]. Curcumin has been shown to have anti-oxidant and anti-inflammatory properties [18] and reduce amyloid plaque burden in transgenic APPsw mice [19]. More recently, curcumin has been shown to reduce the number of aggregates from monomeric Aβ as well as promote disassembly of preformed Aβ aggregates, in addition to inhibiting Aβ oligomer formation and Aβ toxicity at significantly lower concentrations than Ibuprofen [20].

Most *in vitro* functional electrophysiological studies on the effects of Aβ on neurons have been carried out using the patch clamp method [2], [21], [22]. Although the use of this technique enables the acquisition of detailed information concerning Aβ effects at the ion channel level, it is very low throughput and complicated relative to extracellular electrophysiological techniques. A recent technological advance for non-invasive chronic monitoring of neuronal and cardiac cell electrical activity is the use of multielectrode array (MEA) recordings of action potentials [23]–[27]. In contrast to the more common intracellular electrophysiological techniques which usually enable only short term (<a few hours) monitoring of the activity of cells, MEAs are ideal for investigating long-term/chronic drug effects and also does not limit the number of cells that can be recorded from, at a single instance [24], [26], [28]–[30]. Moreover, because MEAs do not require precise positioning of electrodes, they can be used in high-throughput pharmaceutical screens [31]. The most common applications of MEAs include physiological or pharmacological studies in brain slices and in dissociated cell cultures of electrogenic cells including hippocampal neurons [32], [33], spinal cord neurons [34] and cardiac myocytes [24], [25], among others. Recent developments in the pharmacological applications of MEA technology [31] have shown that introduction of high-throughput functional *in vitro* electrophysiological assays in drug development could have significant benefits compared to the traditional *in vivo* or *ex vivo* assays. For example, electrophysiologically active *in vitr*o neuronal networks have been maintained on microelectrode arrays for over 9 months [34]. More recently, neurons on MEAs have been used to study various drug effects including antidepressants [35], ACHe inhibitors [36] and Zn toxicity [37].

In this study we have developed a high-throughput *in vitro* method for the assessment of Aβ effects on spontaneous activity of cultured neurons which can be adapted for high-throughput pharmaceutical screening. This assertion is supported by the emerging view that functional impairment of neurons might be more important for the development of AD symptoms than the actual cell death which occurs at later stages of the disease [1], [2]. The results obtained with MEAs correlate well with those obtained using patch clamp electrophysiology wherein Aβ at low concentrations had a deleterious effect on cell functionality without significant cell death. We have also shown that this effect can be reversed to varying degrees using an anti-amyloidogenic compound. The MEA recording method utilized here is non-invasive, thus long term chronic measurements are possible and it does not require precise positioning of electrodes, thus it is ideal for functional screens. Even more significantly, we believe we have now identified a new target for drug development for AD based on functional toxicity of hippocampal neurons.

## Results

Embryonic rat neurons were plated at a density of 100 cells/mm^2^ on DETA coated coverslips for patch clamp electrophysiology and at 200 cells/mm^2^ on DETA coated microelectrode arrays in serum free medium. Patch clamp electrophysiology was performed after 10 days in culture as electrical function of the neurons had stabilized at this point. Sporadic firing could also be detected after 10 days using the MEAs. Starting on day 12 we were able to obtain stable, reliable recordings from the MEAs over a period of two to three days with an average firing frequency of 2.5±0.6 Hz (mean±SEM). This enabled the study of the time course of the action of low concentrations of Aβ on the neurons. Transferring the MEAs from the incubator to the recording head stage and subsequent media changes did not significantly affect the cells. No significant changes in the baseline recordings from control MEAs were observed as a result of transferring the MEAs from the incubator to the recording headstage or media changes.

### Application of Aβ Abolished Spontaneous Spiking Activity

**Figure 1:**
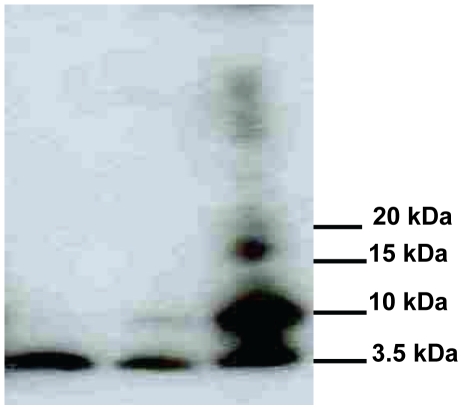
Immunoblot of Aβ oligomers. From left to right: Lane 1 is the monomer. Lane 2 indicates the apparent inhibition of Aβ oligomerization in the presence of Curcumin. Lane 3 indicates Aβ oligomers.

**Figure 2:**
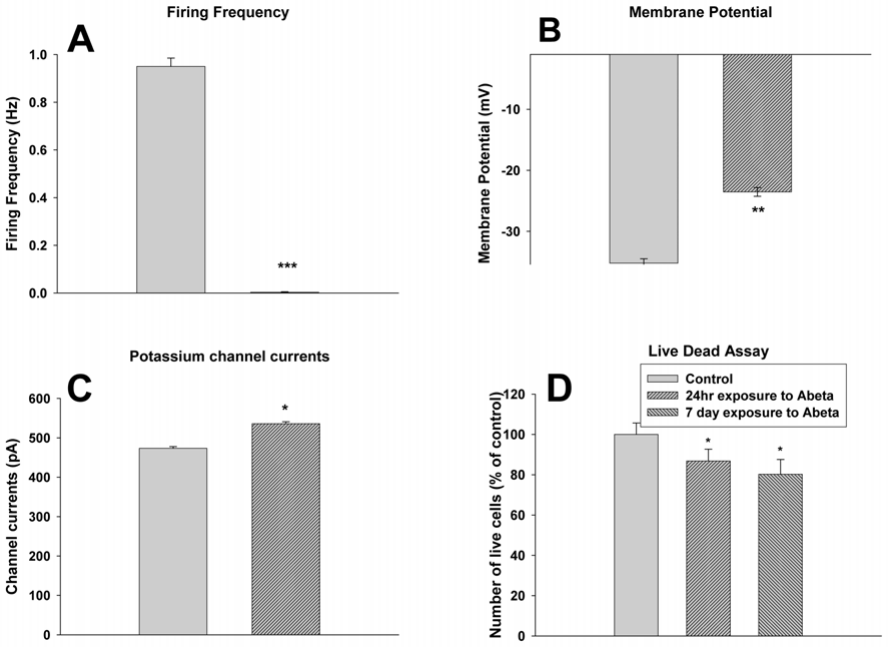
Effect of 24 h exposure to 100 nM Aβ on cell functionality measured using whole cell patch clamp electrophysiology. Changes after exposure to Aβ for 24 hr. in spontaneous firing frequency; ***: p = 0.00007 (A), K^+^ ion channel currents; **: p = 0.008 (B), and membrane potential; *: p = 0.035 (C). Cell survival after administration of 100 nM Aβ at 24 hours and 7 days; *: p = 0.04 (D). Data is presented as mean±SEM; N = 30 utilizing a two-sample Student's t-test.

The presence of Aβ oligomers was verified using immunoblots as shown in Figure 1. Patch clamp experiments performed 24 h post-Aβ exposure revealed striking changes in the neuronal function upon exposure to 100 nM Aβ. The most significant effect was observed on spontaneous firing, namely no spontaneous action potentials were recorded in the 30 exposed cells that were studied at the 24 h time point (Figure *2A*). Exogenous application of Aβ to the cells for 24 h caused an increase in the amplitude of the outward (K^+^) currents as well as a depolarization in the resting membrane potential, (Figure *B,C*). Given the small differences in cell survival compared to the control, even after 7 days (Figure *2D*), we concluded that loss of electrophysiological function is the major response to Aβ treatment at this concentration. To confirm this finding a MTT assay (3-[4,5-dimethylthiazol-2-yl]-2,5-diphenyl tetrazolium bromide) was performed on the amyloid treated cells and this supported the results obtained with the live dead assay.

**Figure 3:**
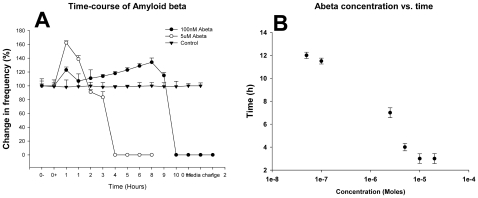
Time course of the effect of Aβ on spontaneous activity of cultured embryonic rat hippocampal cells at various concentrations. Concentrations of 100 nM and 5 µM Aβ caused a complete cessation of firing activity with different time delays (N = 5) (A). Composite logarithmic graph of the time taken for cells to stop firing at the various Aβ concentrations tested (B).

**Figure 4:**
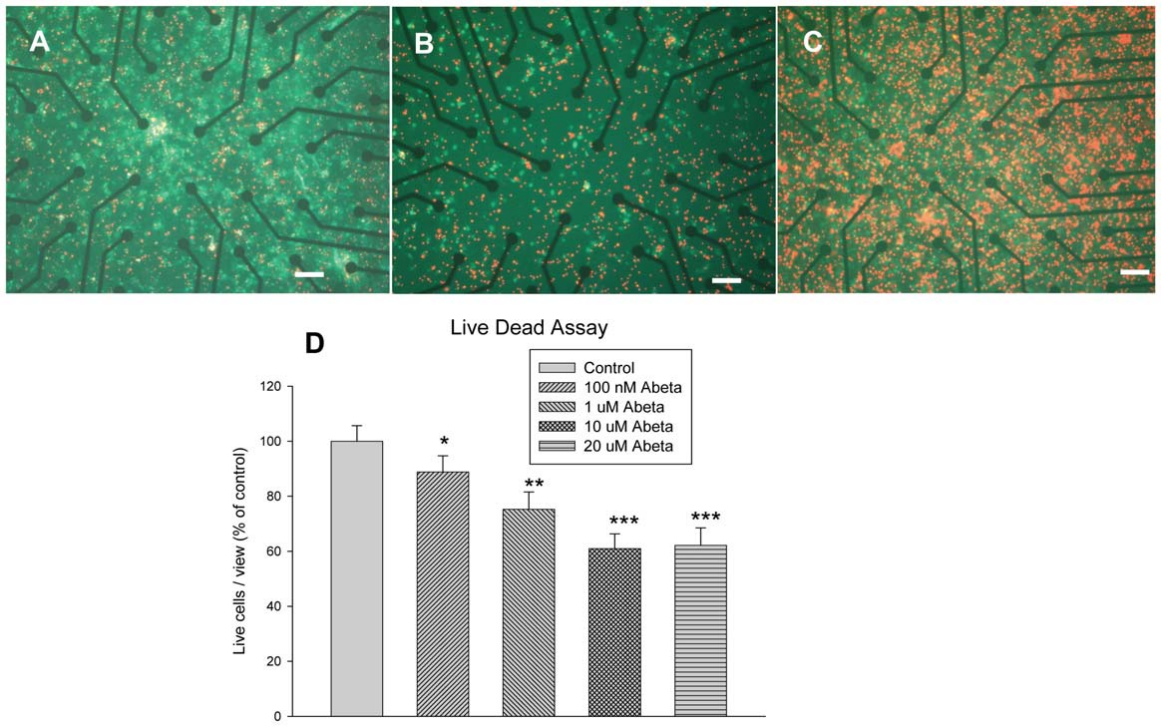
Aβ induced cytotoxicity in hippocampal cells on MEAs. Cell survival before Aβ treatment (A) after treatment with 100 nM Aβ (B) and 20 uM Aβ (C). Green denotes live cells; Red denotes dead cells. Scale bar: 30 µm. Percentage of live cells after treatment with various concentrations of Aβ (D). * p<0.5, **p<0.01, ***p<0.001.

**Figure 5:**
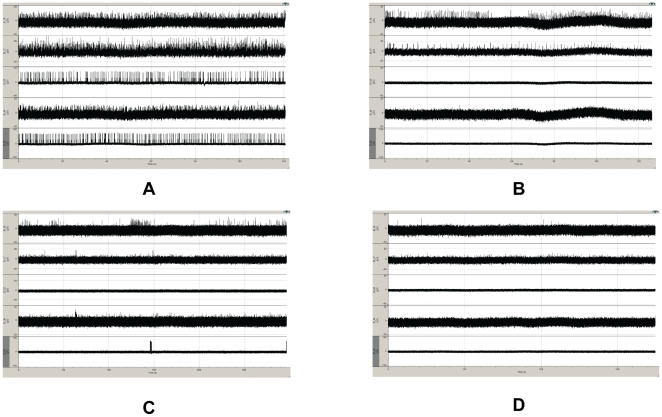
Time course of the application of 20 uM Aβ on spontaneous activity of cultured embryonic rat hippocampal cells on MEAs. Spontaneous firing observed before administration of 20 uM Aβ (A). Spontaneous firing observed 45 minutes after administration of 20 uM Aβ (B), 90 minutes after administration of 20 uM Aβ (C) and 150 minutes after administration of 20 uM Aβ (D).

As a result of the significant effect of Aβ on the firing frequency of the hippocampal neurons, it was decided to use this parameter as a possible new target for implementation in high throughput screens utilizing MEAs. The effect of various concentrations of Aβ on the firing frequency of the neurons on the MEAs that were studied is shown in Figures 3 and 4. At all measured concentrations, Aβ completely abolishes spontaneous spiking activity whereas application of the vehicle control had no effect (Figure 3A). The concentration dependence of Aβ action was quantified by measuring the time required for complete blockade of spiking activity as the dependent parameter in contrast to a more traditional concentration/inhibition relationship. When the cells were exposed to 20 µM Aβ, the highest concentration tested, spiking activity of the cells stopped after about 150 min. At the lowest concentration (50 nM) the cells stopped firing after about 11 h. As seen in Figure *3B*, the time for cessation of spike activity reached a plateau at around 10 µM at the higher end of the concentration range. Blockade of spontaneous activity was preceded by a significant increase in firing frequency at all measured concentrations. At high concentrations of Aβ, high cell death was observed as seen in Figure 4. Figure 5 shows the time course effect of 20 µM Aβ on spontaneous firing frequency of the embryonic hippocampal neurons. These results were in accordance with those obtained using whole cell patch clamp electrophysiology as indicated in Figure 1.

### Partial Functional Activity Could Be Recovered by Administration of Curcumin

**Figure 6:**
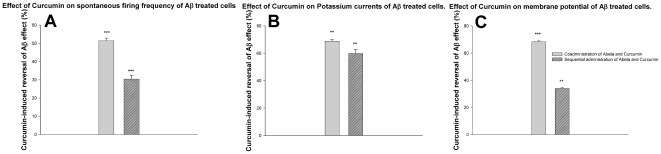
Reversal of the effect of Aβ by curcumin, measured using whole cell patch clamp electrophysiogy. Curcumin was coadminstered with Aβ or sequentially applied after Aβ exposure. The values in Figure 1 were used for baseline comparison. 100% implies complete reversal of Aβ effect and 0% implies no reversal. Effect on firing frequency; ***: p = 0.0007 (A). Effect on potassium currents; **: p = 0.005 (B). Effect on membrane potential; ***: p = 0.0009, **: p = 0.009 (C). Curcumin treated groups were compared with Aβ-only groups using a two-sample Student's t-test. Data is presented as mean±SEM.

In our patch clamp experiments we determined that low doses of curcumin (as previously published) were more successful in ameliorating Aβ toxicity when coadministered with Aβ as opposed to administration after 24 h exposure to Aβ (Figure 6).

**Figure 7:**
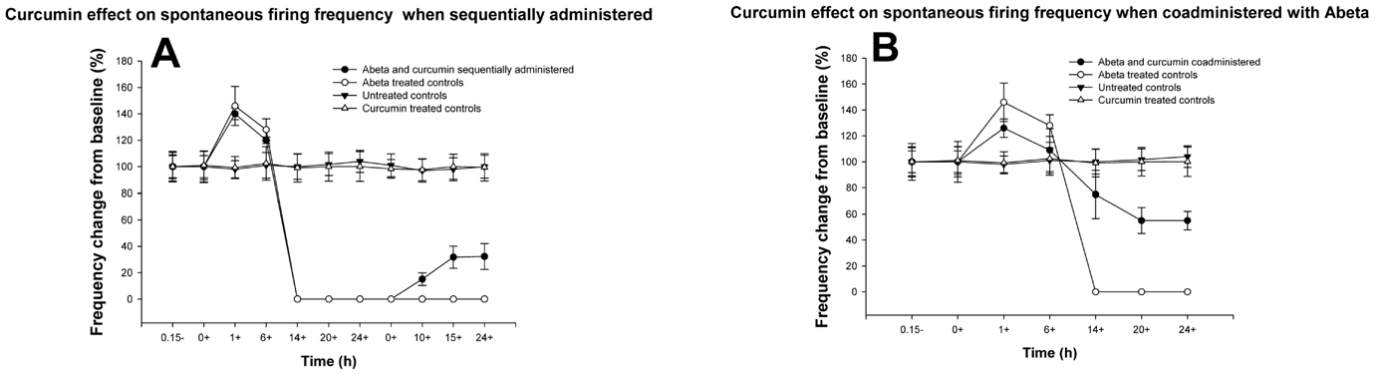
Reversal of the effect of Aβ on firing frequency by curcumin applied together with Aβ or after Aβ exposure. Time course of the effect of curcumin on spontaneous firing frequency of embryonic hippocampal neurons when coadministered with Aβ (N = 5) (A). Time course of curcumin effect on spontaneous firing frequency of embryonic hippocampal neurons when administered after cells were exposed to Aβ for 24 h (N = 5) (B). 100% implies baseline values before exposure to Aβ.

Thus, having demonstrated that Aβ functional toxicity could be reproduced using multielectrode arrays, a screening assay was then demonstrated by measuring the recovery of the lost functionality using the anti-amyloidogenic compound curcumin. Based on the control methodology in Figure 6, two modes of curcumin application were used. Curcumin was coadministered with Aβ to the cells on the MEAs for a 24 hour period. In the second set of experiments curcumin was applied sequentially after the cells were exposed to Aβ for 24 hrs. We observed that functional recovery as recorded by the MEAs was similar to the patch-clamp experiments. As seen in Figure 7A, when curcumin was coadministered with Aβ, the cells were able to maintain 54.9±0.7% (mean±SEM) of their baseline firing activity, as opposed to a complete loss of functionality when treated with Aβ alone. The decline in firing frequency was more gradual and the drop in firing frequency reached a plateau about 18 hrs after curcumin and Aβ were coadministered. Administration of curcumin after the cells were exposed to Aβ for 24 hrs resulted in a gradual recovery of firing frequency to 29.9±0.7% (mean±SEM) of the baseline (Figure 7B). In this paradigm, recovery of spontaneous firing was observed around 10 hrs after curcumin was applied post Aβ exposure. The recovery of spontaneous firing frequency obtained with curcumin treatment was comparable to results obtained with patch clamp electrophysiology using similar experimental paradigms, as shown in Figure 6.

## Discussion

Our initial results using whole-cell patch clamp electrophysiology demonstrated that Aβ affects electrical functionality earlier and at lower concentrations than which affect the survival of the cells. It is possible this effect could also precede synapse degradation or that it may be its upstream cause. Previous results had hinted at this idea, for example Chen and coworkers reported that various low concentrations of Aβ inhibited long-term potentiation (LTP) in hippocampal slices [2], [38], [39]. Based on these results, Ahuja et al. used MEA technology to measure Aβ effect on LTP in organotypic hippocampal cultures [40]. The importance of these investigations is highlighted by the significant need in the pharmaceutical industry for an *in vitro* model of the early stages of Alzheimer disease and the functional effects of Aβ on neurons observed in the study might be considered as an *in vitro* AD model.

We then utilized this result to create a high-throughput screening method for antagonists of this functional toxicity caused by Aβ. The MEAs made it possible to screen a significantly higher number of cells for Aβ and drug effects in a much shorter amount of time than patch-clamp electrophysiology would have required. Development of this method could have a high impact on drug development in Alzheimer's disease (AD). The molecular target of Aβ toxicity is not well known, thus this functional screen could result in novel effective compounds or therapeutic targets. We have shown that multielectrode arrays (MEAs) can be used to reliably detect functional effects of low doses of Aβ (100 nM) as well as screen for the rescue effect of curcumin. When applied to hippocampal neurons cultured on MEAs Aβ had a pronounced effect on the spontaneous firing of the cells, even at concentrations in the nanomolar range. Treatment with Aβ stopped spontaneous activity completely and the time for cessation was concentration dependent. The Aβ oligomerization inhibitor, curcumin, was able to partially reverse the loss of spontaneous activity. In accordance with our earlier patch clamp experiments, curcumin was more effective in inhibiting the effect of Aβ when it was coadministered with it as opposed to the experiments in which it was applied 24 hrs after Aβ exposure.

Interestingly, after Aβ exposure, there was a slight but consistent increase in firing frequency just before the decline of spontaneous activity. The initial increase in firing frequency we observed at all tested Aβ concentrations could be due to an earlier reported direct depolarizing effect of Aβ on the membrane potential or to the reputed ability of Aβ to enhance glutamate-mediated excitotoxicity [41], [42] by its action on NMDA receptors and consequently, through an increased influx of Ca^2+^.

In comparison to slice preparation, our method, measurement of the effect of Aβ on spontaneous activity of cultured neurons, is significantly simpler and more applicable in high-throughput screen methodology. Another benefit of this MEA AD model, compared to our patch-clamp experiments, was that we were able to follow the time course of the action of curcumin on the Aβ modified activity of the same population of cells. When Aβ and curcumin were applied together, curcumin reduced the deleterious effect of Aβ without a significant change in the time course of Aβ action (Figure *5*A). When Aβ and curcumin were applied sequentially, curcumin reversed the effect of Aβ and helped the cells to partially recover their spontaneous firing activity (Figure *5*A). Curcumin was more effective when administered together with Aβ; the cells were able to retain about 55% of their firing capability compared to untreated controls when coadministered as opposed to only 30% when sequentially administered. It has been shown that curcumin was able to inhibit Aβ oligomer formation and reduce amyloid toxicity *in vitro* [20]. In the presence of curcumin, reduced aggregation from monomeric Aβ and improved disassembly of preformed Aβ aggregates was observed [20]. Curcumin's ability to disassemble pre-formed Aβ aggregates could account for its protective effect against Aβ toxicity in the co-administration experiments, but the mechanism involved in the reversal of Aβ toxicity in the post-administration experiments needs further clarification.

In conclusion, this study demonstrated that it is possible to develop a high-throughput screen for the measurements of drug effects on functional toxicity of low concentrations of Aβ and this model might be considered as an *in vitro* functional model of the development of Alzheimer's disease. This screen method, based on MEA technology, which enables the screening of a large number of cells, and the study of pathogen and drug effects on the same population of cells over an extended period of time, could find important applications in pharmaceutical drug development and could lead to novel drug candidates or therapies for AD. Moreover, based on similar principles, MEA technology can be potentially extended to study in vitro models of other neurodegenerative diseases as well.

## Materials and Methods

### Microelectrode Arrays

The MEAs and accompanying accessories, including the temperature controller, stimulator, amplifier and MC_Rack V 3.5.8 data acquisition software were obtained from ALA Scientific (Westbury, New York) and Multichannel Systems (Reutlingen, Germany). The MEAs comprised of a glass base that acted as a substrate, gold connector contacts and electrodes composed of titanium nitride. Rings were made of Sylgard184 (Dow Corning) (1 part curing base and 10 parts elastomer base, cured at 60°C for 45 minutes) using glass molds and were attached onto the MEAs after surface modification.

Recordings were obtained from 12–16 D. old cultures. Cultures were kept in the incubator between recording sessions.

### Surface Modification

*N*-1(3-[trimethoxysilyl]propyl)-diethylenetriamine (DETA) was used to modify the MEAs to enhance cell attachment since the use of synthetic substrates such as DETA, allows for reproducible and precise quantification of the culture substrate properties [43]. Glass coverslips (18 mm diameter, Number 1; VWR) were cleaned in two steps. First, they were soaked in 50/50% HCl (37%) (VWR)/methanol (Sigma), followed by H_2_SO_4_ (98%) (VWR) treatment. Next, they were rinsed in double distilled water. The coverslips were then boiled in deionized water, rinsed with acetone, and oven dried. The MEAs were initially cleaned overnight in 2% Tergazyme (Sigma) detergent solution. They were then rinsed in distilled water and plasma cleaned in a plasma cleaner (Harrick Plasma) for 30 mins. The *N*-1(3-[trimethoxysilyl]propyl)-diethylenetriamine (DETA) (United Chemical Technologies) surface assembled monolayer (SAM) film was formed by the reaction of the cleaned surfaces with a 0.1% (v/v) mixture of the organosilane in toluene. The DETA/toluene solution containing the MEAs was heated to 70°C, rinsed with toluene, reheated to 70°C, and then oven dried. Surfaces were characterized using contact angle measurement and X-ray photoelectron spectroscopy (XPS) as described previously [44].

### Cell Culture

All applied procedures were approved by the Institutional Animal Care and Use Committee of UCF. The protocol was modified from previously published work concerning embryonic rat hippocampal cultures [43], [45]. Pregnant rats, 18 days in gestation, obtained from Charles River were euthanized with carbon dioxide and the fetuses were collected in ice cold Hibernate E (BrainBits)/B27/Glutamax™/Antibiotic-Antimycotic (Invitrogen) (dissecting medium). Each fetus was decapitated and the whole brain was transferred to fresh ice cold dissecting medium. After isolation, the hippocampi were collected in a fresh tube of dissecting medium. Hippocampal neurons were obtained by triturating the tissue using a Pasteur pipette. In order to remove any debris from damaged cells the 1 ml cell suspension was layered over a 4 ml step gradient (Optipep diluted 0.505: 0.495 (v/v) with the dissecting medium and then made to 15%, 20%, 25% and 35% (v/v) in the dissecting medium) followed by centrifugation for 15 min at 800 g and 4°C. After centrifugation, one strong band of cells was obtained. This band of cells was resuspended in culture medium (Neurobasal/B27/Glutamax™/Antibiotic-Antimycotic) and plated at a density of 100 cells/mm^2^ on DETA coated coverslips for patch clamp electrophysiology and at 200 cells/mm^2^ on the MEAs.

### Aβ (1–42) and Curcumin Administration and Electrophysiology

Different concentrations of Aβ (1–42) (Bachem) aggregates were prepared according to the protocol by Klein [1] in Neurobasal medium without phenol red, and quantified using Immunoblots. Curcumin (Cayman Chemicals) was prepared and the concentration chosen according to previously published protocols [20].

For patch clamp electrophysiology experiments, Aβ was administered to the cells on day 10 *in vitro* and recordings were performed after 24 hrs to obtain baseline values for control cells and Aβ treated cells. In coadministration experiments, a mixture of 100 nM Aβ and 1 µM curcumin was administered for 24 hrs after which patch clamp electrophysiology recordings were performed. In sequential administration, cells were initially exposed to freshly aggregated Aβ alone, followed by replacement of the Aβ solution with curcumin for 24 hrs patch clamp recordings were performed 24 hrs after curcumin treatment. Whole-cell patch clamp recordings were performed at room temperature in a recording chamber on the stage of a Zeiss Axioscope 2 FS Plus upright microscope as described in [44].

In all experiments with MEAs Aβ was administered to the cells on day 16 *in vitro*. We chose experimental paradigms similar to those used for patch clamp electrophysiology, to study the potential therapeutic effects of curcumin. In coadministration experiments, cells were exposed to a mixture of 100 nM Aβ and 1 µM Curcumin for 24 hrs, and recordings were performed. In the second paradigm, cells were initially exposed to freshly aggregated Aβ alone, followed by replacement of the Aβ solution with curcumin for 24 hrs. Extracellular recordings were obtained before the administration of Aβ, immediately after Aβ administration, 24 h after Aβ administration, before curcumin administration and 24 hrs after curcumin administration.

### Cytotoxixity Assays

Using the LIVE/DEAD™ viability/cytotoxicity kit (Molecular Probes), and the MTT based *in vitro* toxicology assay kit (Sigma), cell survival was measured as per the instructions that accompanied the kits.

### Data Analysis and Statistical Methods

Frequency values for all data points on MEAs were averaged over 5 MEAs. For patch clamp electrophysiology, a sample size of N = 30 was used. Changes in parameters induced by all externally applied chemicals were quantified as a percentage of baseline values. Statistical significance was calculated used students t test.
